# Premature Decay of Our Teeth

**Published:** 1879-08

**Authors:** 


*Premature Decay of Our Teeth.*—Geo. M. Beard, M. D., of New York, in the article on "American Nervousness," takes the ground in reference to the premature decay of American teeth, that the cause is subjective rather than objective—is in the constitution of modern civilized man. A doctrine undoubtedly correct. The dentist of the present is a necessity, growing not out of our habits, but out of our climatic influences and constitutional developments, and will, in the future, be still more a necessity. H.

